# Scientific News

**Published:** 1880-03

**Authors:** 

**Affiliations:** Keene, N. H.


### Scientific News.

For some years past there has been a decided opposition to the doctrines of evolution upon the part of eminent divines, both as teachers in educational spheres and as church men merely. The resistance is observed to be more decided and pronounced amongst the older clergy than the younger, the latter have been and are being educated in a more liberal manner, and it is now becoming a recognized fact that there is nothing inherently wicked in the teachings of modern science. There is abundant evidence to show that the researches of science are merely for the acquisition of knowledge and the higher development of the human mind. It is certainly gratifying and is indicative of a future harmony between men pursuing different lines of thought, that, the budding theologian is being nourished with something solid, and the better fitted to take a comprehensive view of what is being done in the domain of mind.

The following letter to the *Popular Science Monthly* for March, explains itself :

Messrs. Editors,

In a good but caustic review of Mr. Mallock's book, "Is Life Worth Living?"—you make use of a sentence which would seem to reflect on all alike who are engaged in the study of theological problems; "we have here the last brilliant exploit of the theological mind in its warfare with modern science." Permit me, as a student of theology and a lover of modern science, to read you a short lecture: many of the young ministers to-day are firm believers in evolution and preach it. This theory is by no means a hinderance in our study of theology, but the best instrument which has so far been placed in our hands.

If on our desk the Bible lies, so also do Spencer's "First Principles" and his "Sociology." If we respect and study Jesus, so do we Spencer and Tyndall and Clark Maxwell, these have a gospel for us—they have a hope, the young theological mind is very far from engaged in a warfare with science; it is anxious and hoping for firmer ground than we now have, if science can help us, and it can and does in making this life more valuable, the future brighter, and ourselves better, we welcome it. We are not troubled about reconciling theology and science; we take what we can in both, after honestly and carefully investigating for ourselves, and then allow them to reconcile themselves. What we have to do with is the truth, pure and simple. Some of us will not pledge ourselves to any "body of divinity," either ancient or modern; we will not swear by Bibles, old or new, nor believe all the spirits, either in the Gospels or the biologies.

The writer of the preface to the American Edition of Spencer's "First Principles," tells us that his hope is in the young men, many of them are with him. We now only ask you to remember this, and let us investigate in our own fields, mindful of the fact that we are each doing our best to find the truth, we are side by side oftener than we imagine, even if some college presidents will not see it, none are so blind as those who will not see, it would not be out of place if in the "Monthly," you would give an article now and then, bearing *directly* on the theological questions, not the petty disputes of the sects.

You have our hand, A YOUNG THEOLOGIAN.

KEENE, N. H., January 21st, 1880.

